# Electrical Reverse Remodeling of the Native Cardiac Conduction System after Cardiac Resynchronization Therapy

**DOI:** 10.3390/jcm9072152

**Published:** 2020-07-08

**Authors:** Hee-Jin Kwon, Kyoung-Min Park, Seong Soo Lee, Young Jun Park, Young Keun On, June Soo Kim, Seung-Jung Park

**Affiliations:** Division of Cardiology, Department of Internal Medicine, Heart Vascular and Stroke Institute, Samsung Medical Center, Sungkyunkwan University School of Medicine, 81 Irwon-ro, Gangnam-gu, Seoul 06351, Korea; subakhj@gmail.com (H.-J.K.); bkm1101@hanmail.net (K.-M.P.); smclupin@gmail.com (S.S.L.); youngjun.park@samsung.com (Y.J.P.); yk.on@samsung.com (Y.K.O.); js58.kim@samsung.com (J.S.K.)

**Keywords:** cardiac resynchronization therapy, electrical reverse remodeling, PR interval, QRS duration, heart failure

## Abstract

**Background:** Little is known about electrical remodeling of the native conduction systems, particularly how the PR interval changes, after cardiac resynchronization therapy (CRT). We investigated the effects of CRT on the intrinsic PR interval (i-PRi) and QRS duration (i-QRSd). **Methods and results:** In 100 consecutive CRT recipients with sinus rhythm and long-term follow-up (>1 year), the i-PRi and i-QRSd were measured at baseline and at the last echocardiographic follow-up (33.4 ± 17.9 months) with biventricular pacing temporarily withdrawn. The relative decrease in the left ventricular end-systolic volume (LVESV) was measured to define CRT-responders (≥15%) and super-responders (≥30%). Following CRT, the left ventricular (LV) ejection fraction increased significantly (p < 0.001). In CRT-responders (n = 71), the LVESV and i-QRSd decreased markedly (170 ± 39 to 159 ± 24 ms, p = 0.012). However, the i-PRi was not shortened with CRT response and was actually likely to increase, even in the super-responder group (n = 33). Moreover, lengthening of the i-PRi was observed consistently irrespective of the CRT response status, beta-blocker use, or amiodarone use. CRT non-responders were associated with a remarkable PR prolongation (p = 0.005) and QRS widening (p = 0.001), along with positive ventricular remodeling. **Conclusion:** LV volume and i-QRSd decreased markedly with CRT response. However, the i-PRi was not shortened, but rather increased regardless of the degree of CRT response. CRT non-response was associated with a considerable increase in the i-PRi and i-QRSd, along with positive ventricular remodeling. CRT-induced electrical reverse remodeling might occur preferentially in the intraventricular, but not the atrioventricular, conduction system.

## 1. Introduction

Cardiac resynchronization therapy (CRT) is an important treatment option for advanced heart failure (HF) patients with wide QRS complex [1]. Structural reverse remodeling with CRT has been well demonstrated in many studies, which have shown that it reduced the left ventricular (LV) chamber size and significantly improved LV systolic function [2]. The degree of that improvement was directly related to the likelihood of overall death or HF-related hospitalization (HF-hospitalization) [2]. However, electrical reverse remodeling has not been carefully assessed. Previously, some studies have shown that CRT predictably decreased paced QRS duration (p-QRSd) and even intrinsic QRSd (i-QRSd), and that reduction of the i-QRSd and p-QRSd was closely associated with better clinical and echocardiographic response to CRT [3,4]. However, the number of patients in those studies was very small. Moreover, few studies have been conducted on changes in the intrinsic PR interval (i-PRi), which represents the atrioventricular (AV) conduction property, following CRT.

Prolonged PRi is generally considered a benign disease. However, several previous studies have demonstrated that PR prolongation correlates with worse prognosis in the general population and HF patients with and without CRT implantation [5,6]. Although no causal relationship between abnormal PRi and worse clinical outcomes has been fully elucidated, PRi can significantly affect hemodynamics. A short PRi causes truncation of mitral A-waves [7,8]. In contrast, prolonged PRi leads to the fusion of mitral E- and A-waves and diastolic mitral regurgitation [7,8]. Both short and prolonged PRi decrease LV diastolic filling, and eventually result in low cardiac output. PR prolongation was found in about 40% of CRT patients, a much higher frequency than found in the general population. Furthermore, optimization of the AV delay, a counterpart of PRi for CRT devices, is one of the most important aspects in the management of CRT patients [8,9]. Recently, several automated algorithms designed to optimize the AV or interventricular (VV) delay, such as SyncAV (Abbott, Sylmar, CA, USA), SonR (Sorin Group, Saluggia, Italy), and the AdaptivCRT algorithm (Medtronic Inc., Minneapolis, MN, USA) have been adopted to improve CRT response [10,11,12]. However, to apply those automated optimization algorithms, the i-PRi needs to be maintained within a relatively short range (<220~250 ms). In patients with prolonged PRi, these algorithms could not be applied. Then, we hypothesized that shortening the i-PRi through electrical reverse remodeling following CRT, would allow those algorithms to be applied to more patients. Therefore, in this study, we investigated the effects of CRT on the native conduction system, with a particular focus on changes in the i-PRi and i-QRSd.

## 2. Materials and Methods

### 2.1. Study Population

Clinical, electrocardiographic (ECG), echocardiographic (Echo), and CRT-related variables in all patients undergoing CRT implantation at our hospital are gathered prospectively in our CRT registry. We screened all consecutive patients with a CRT implantation between January 2010 and June 2018. The inclusion criteria for this study were patients who (1) were older than 18 years, (2) had sinus rhythm, and (3) were followed up more than 1 year after the CRT implantation. Patients were excluded if they had (1) an unmeasurable i-PRi due to ongoing atrial fibrillation (AF) or AV block, (2) a ventricular pacing rhythm before CRT implantation, or (3) missing ECG or Echo data at baseline or during follow-up. Baseline and follow-up data including demographics, medications, and ECG and Echo variables were collected through a careful review of our CRT registry and electronic medical records. All of the enrolled patients also had the following characteristics, consistent with standard guidelines: New York Heart Association functional class II, III, or ambulatory IV congestive HF, QRSd ≥ 120 ms, and LV ejection fraction (EF) ≤ 35%. The study was approved by the Institutional Review Board of Samsung Medical Center and the requirement of written informed consent was waived (IRB No. 2020-03-076).

### 2.2. Measurement of ECG Variables

Standard 12-lead ECGs (25 mm/s, 10 mm/mV) were taken before and after CRT implantation to collect various ECG data, including heart rate (HR), paced/intrinsic PRi and QRSd, and QRS morphology. At the regular 3-month device follow-up appointment, 12-lead ECGs were taken in the supine position after about 15 min of rest to minimize the effect of autonomic tone on the intrinsic ECG variables at our dedicated device clinic. First, ECGs were obtained during biventricular pacing. Next, intrinsic ECG rhythms were recorded again after patients’ intrinsic rhythms were allowed to continue at least one or two minutes by lowering the pacing rate to 30 beats/minute in ventricular demand pacing (VVI) mode. In this study, we used ECG tracings that were chronologically closest to the follow-up Echo to analyze changes in the intrinsic PRi and QRSd. The PRi was measured from the beginning of the P-wave deflection to the onset of the QRS complex, the QRSd was measured from its first deflection (or from the pacing artifact for p-QRSd) to its end (J point). The paced/intrinsic PRi and QRSd were manually measured using electronic calipers (CIS Viewer Version 2.0.1.4; INFINITT Healthcare, Seoul, Korea) by an experienced cardiologist who was blind to CRT response status. Baseline QRS morphology was divided into either left bundle branch block (LBBB) or non-LBBB.

### 2.3. Echocardiographic Data

Transthoracic Echo was performed before and after CRT implantation using commercially available equipment (Vivid 9 or 7 from GE Healthcare, Chicago, IL, USA; Sonos 5500 from Philips, Andover, MA, USA; or Acuson 512 from Siemens, Munich, Germany). LV end-diastolic volume (LVEDV) and end-systolic volume (LVESV) were determined by semiautomatic tracing of the end-systolic and end-diastolic endocardial borders using apical four-chamber and two-chamber views. LV EF was calculated with the biplane modified Simpson’s method. Patients who showed a relative reduction in LVESV <15% were considered to be non-responders to CRT. Regular responders were defined as patients with a relative reduction in LVESV between 15% and 30%, and super-responders were those with a relative reduction in LVESV of ≥30% and an LV EF ≥45% in a follow-up Echo [12].

### 2.4. Statistical Analysis

Data are presented as numbers and frequencies for categorical variables and as mean ± standard deviation (SD) for continuous variables. For comparisons between groups, the χ2 test or Fisher exact test was performed for categorical variables, and the student’s t-test or Mann–Whitney test was used for continuous variables. A paired *t*-test or Wilcoxon signed rank test was performed appropriately to compare differences between the pre- and post-CRT ECG and Echo variables. Scatter-plots of the ECG and Echo variables were constructed, and a Pearson’s correlation analysis was performed to evaluate the correlations between the electrical and mechanical reverse remodeling. A *p*-value of <0.05 was considered statistically significant. All statistical analyses were performed using SPSS version 25.0 (IBM., Armonk, NY, USA).

## 3. Results

### 3.1. Study Population and Design

**Figure 1:**
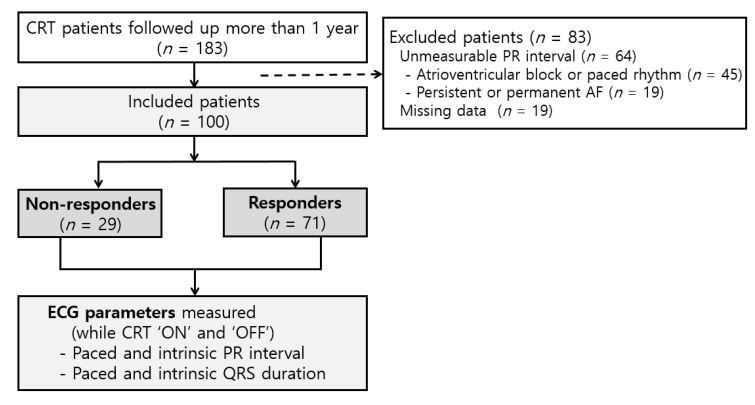
Flow chart of the study population.

Between January 2010 and June 2018, we identified 183 CRT patients with more than one-year of follow-up. Of the 183 CRT patients, 83 were excluded for AV block or ventricular paced rhythm (*n* = 45), persistent/permanent AF (*n* = 19), or missing ECG/Echo data (*n* = 19) (Figure 1). The remaining 100 patients were included in our final analyses. The mean (±SD) age of our patients was 65.7 ± 11.5 years and 30 patients had ischemic causes of HF. Baseline LV systolic functions were severely depressed in all patients, with a mean (±SD) LV EF of 24 ± 6%. The mean (±SD) values of intrinsic QRSd and PRi were 166 ± 36 ms and 199 ± 34 ms, respectively. LBBB morphology was observed in 80 patients.

**Figure 2:**
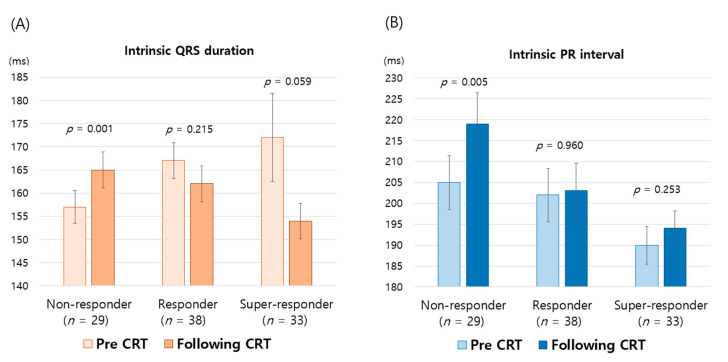
Changes in the native QRS duration (**A**) and PR interval (**B**) according to the degree of cardiac resynchronization therapy (CRT) response.

During the mean Echo follow-up period of 33.4 ± 17.9 months, 29 patients were classified as non-responders, and 71 fit into the responder groups according to the predefined responder criteria (Figure 1, Table 1). Of the 71 responders, 38 patients showed a regular response, and the remaining 33 were super-responders to CRT (Figure 2). The CRT responders had a lower proportion of hypertension (*p* = 0.023) and ischemic cardiomyopathy (*p* = 0.023) than the non-responders (Table 1). LV dimensions were less severely dilated in the responder group than the non-responder group. LBBB morphology was more frequently observed in responders than non-responders (*p* = 0.004). However, the baseline HR, QRSd, PRi, and corrected QT interval did not differ significantly between responders and non-responders. At discharge, beta-blockers were more frequently prescribed to the responder groups (*p* = 0.072), whereas the non-responder group received more amiodarone (*p* = 0.030).

ACE inhibitor, angiotensin converting enzyme inhibitor; ARB, angiotensin receptor blocker; LVEF, left ventricular ejection fraction; LVEDV, left ventricular end-diastolic volume; LVESV, left ventricular end-systolic volume; QTc interval, corrected QT interval.

### 3.2. Structural Reverse Remodeling after CRT Implantation

After CRT implantation, the mean (±SD) LV EF of the study population improved greatly at the last Echo follow-up (33.4 ± 17.9 months), from 24 ± 6% to 40 ± 15%, *p* < 0.001 (Table 2). Remarkable reductions in LVEDV (251 ± 66 to 183 ± 89 mL, *p* < 0.001) and LVESV (192 ± 58 to 122 ± 82 mL, *p <* 0.001) were also observed (Table 2). Structural reverse remodeling was primarily achieved in the responder groups, whose absolute reduction in LVEDV and LVESV measured 101 ± 58 and 103 ± 42 mL, respectively (Table 3). In contrast, the LV dimension in the non-responder group dilated further (LVEDV by 15 ± 38 mL, LVESV by 10 ± 30 mL) indicating positive structural remodeling (Table 3).

### 3.3. Electrical Reverse Remodeling after CRT Implantation

#### 3.3.1. Change in the Intrinsic QRS Duration and PR Interval

The i-QRSd, which was measured during the CRT-off mode, decreased significantly in the responder group: 170 ± 39 to 159 ± 24 ms, *p* = 0.012 (Table 2). In contrast, the i-QRSd became more extended in the non-responder group (157 ± 19 to 165 ± 20, *p* = 0.001). Therefore, when the study population was divided into three groups, non-responders (*n* = 29), regular-responders (*n* = 38), and super-responders (*n* = 33), a clear trend toward a greater reduction in the i-QRSd with the degree of CRT response became evident (Figure 2A).

Unlike the i-QRSd, the i-PRi was not shortened with CRT response (Table 2). On the contrary, the i-PRi was likely to increase further even in the super-responder group (Figure 2B), though the increase was more pronounced in the non-responder group. Additionally, lengthening of the i-PRi was observed consistently irrespective of the CRT response status, beta-blocker use, or amiodarone use (Table 2 and Table 4). However, the proportions of patients taking beta-blockers or amiodarone at the last follow-up were almost comparable to those at discharge: from 83% to 87%, *p* = 0.553 for beta-blockers; from 18% to 17%, *p* = 1.000 in amiodarone (Table 4).

On the other hand, p-QRSd also decreased greatly, from a baseline of 166 ± 36 ms to 134 ± 21 ms (*p* < 0.001) after CRT. The amount of decrease was greater in the responder than non-responders: 40 ± 42 vs. 12 ± 27 ms, *p* = 0.002 (Table 3).

#### 3.3.2. Correlation between Structural and Electrical Reverse Remodeling

**Figure 3:**
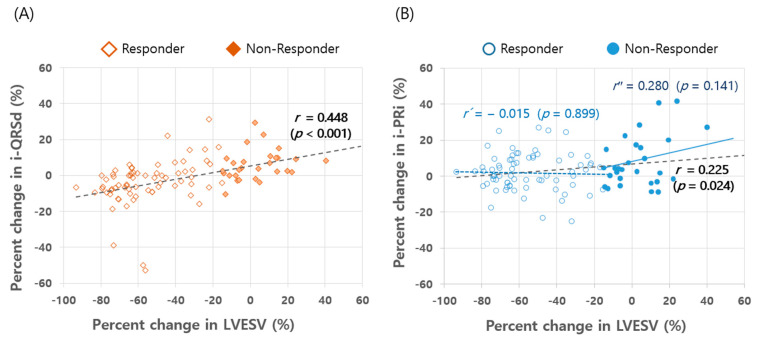
Correlation between relative changes in LV volumes and intrinsic QRSd (**A**) and PR interval (**B**). Responders and non-responders to CRT were divided by −15% in relative change in LVESV. LVESV, left ventricular end systolic volume; i-PRi, intrinsic PR interval; i-QRSd, intrinisic QRS duration.

To evaluate the relationship between structural and electrical reverse remodeling, we tested the correlation between percent changes in the LVESV and i-QRSd or i-PRi. A moderately strong positive correlation was found between percent reductions in the LVESV and the i-QRSd (*r* = 0.448, *p* < 0.001) (Figure 3A). In contrast, the relative change in i-PRi showed only a weak positive correlation with the relative change in LVESV as a whole (broken black line, *r* = 0.225, *p* = 0.024) (Figure 3B). However, in the responder groups (*n* = 71), the significant correlation between the relative changes in the LVESV and i-PRi disappeared (broken blue line, *r*′ = −0.015, *p* = 0.899). In the non-responder group, the i-PRi was likely to increase further with LVESV enlargement (solid blue line, *r*″ = 0.280, *p* = 0.141) (Figure 3B).

## 4. Discussion

### 4.1. Main Findings

Our purpose in this study was to investigate electrical reverse remodeling following CRT by comparing ECG parameters before and after CRT implantation. We focused primarily on changes in the i-PRi and i-QRSd according to the level of CRT response. Our main findings are that LV volumes were significantly reduced and LV EF improved markedly with CRT response, as shown in previous studies [2]. In terms of electrical reverse remodeling, i-QRSd, measured during CRT-off mode, decreased significantly in the CRT-responder group. However, there was no shortening of i-PRi even in the super-responder group. Contrary to our expectations, i-PRi tended to increase irrespective of the level of CRT response. CRT non-response was associated with a remarkable PR prolongation (*p* = 0.005), and QRS widening (*p* = 0.001), and positive ventricular remodeling (Figure 2 and Figure 3).

### 4.2. Changes in PR Interval and QRS Duration Following CRT

Several previous studies demonstrated that CRT induced both electrical and structural reverse remodeling, with the former mostly represented by narrowing of the i-QRSd [2,3,4,13,14,15]. In those results, CRT responders showed a significant decrease in their i-QRSd during the follow-up, in addition to the immediate reduction in p-QRSd achieved by biventricular pacing [3,4,13]. In a prospective study of 85 CRT patients, Sebag et al. reported that shortening of the i-QRSd ≥ 20 ms was associated with better clinical and echocardiographic response at the one-year follow-up [3].

On the other hand, few studies have investigated change in the i-PRi after CRT implantation. The clinical implications of prolonged PRi were evaluated in a sub-study of the Cardiac Resynchronization-Heart Failure (CARE-HF) trial: a longer PRi observed at baseline and at 3 months post-CRT predicted all-cause mortality and HF-hospitalization during follow-up. However, the authors measured 3-month PRi during biventricular pacing, so information on the i-PRi change could not be retrieved from that sub-study [15]. Two previous studies presented data on i-PRi changes, but they evaluated cohorts of only 25 and 21 patients, respectively [13,14]. Additionally, they did not analyze the i-PRi change depending on the degree of CRT response. In the study of 25 patients, i-PRi increased from 193 ± 57 to 198 ± 52 ms at 14 months post-CRT (*p* > 0.2), and in the other study (*n* = 21) it lengthened significantly at 21 months (175 ± 29 to 188 ± 35 ms, *p* = 0.03). Interestingly, our results correspond well with the former results: the i-PRi increased regardless of the degree of CRT response and increased significantly in CRT non-responders (Figure 2B). However, as easily expected, there was a good correlation between the degree of CRT response and the amount of QRS narrowing (Figure 2A and Figure 3A). Therefore, our findings suggest that CRT-induced electrical reverse remodeling might occur preferentially in the intraventricular conduction system, but without remarkably affecting the AV conduction system.

The underlying mechanisms explaining why i-PRi and i-QRSd show differential responses to CRT remain unclear, but several possibilities can be offered. First, the QRSd reflects the total duration of ventricular activation and correlates positively with LV dimensions in patients with cardiomyopathy both with and without LBBB [16]. Therefore, shortening of the i-QRSd might be caused in part by structural reverse remodeling of the ventricles, and indeed, we observed a statistically significant correlation between percent reductions in the two variables (*r* = 0.448, *p <* 0.001, Figure 3A). However, volumetric change in the AV node was probably negligible compared to the prominent ventricular structural remodeling after CRT. Therefore, the i-PRi, which reflects mainly the conduction time in the AV node, would not be significantly affected by the volumetric change in the AV node or the ventricles; in our results, the percentage change in the i-PRi correlated only weakly with the degree of change in the LVESV, unlike the i-QRSd (Figure 3A,B). Secondly, anatomical difference in cardiac autonomic innervation between supraventricular structures (sinoatrial or AV nodes and atria) and ventricular muscles, may be also involved. HF-induced autonomic imbalance, that is, sympathetic overactivity and parasympathetic withdrawal, can be alleviated or reversed in CRT responders [17,18]. Then, atria and nodes are more heavily innervated by autonomic nerves than ventricular muscles [17]. Therefore, the effect of restored sympathovagal balance by CRT (decreased sympathetic tone and restored parasympathetic activity) might be more accentuated in the atria and nodes than in the ventricles, which might contribute to the prolongation of the i-PRi even in CRT responders. Next, in a similar context, i-QRSd generally remains almost constant irrespective of HR, whereas i-PRi shows an inverse correlation with HR during exercise testing [19,20]. In our results as well, HR change inversely correlated only with the i-PRi change (*r* = −0.227, *p* = 0.023), with changes in the i-QRSd and HR having no significant correlation (*r* = −0.063, *p* = 0.536). Therefore, CRT-induced improvement in HF symptoms, LV functions, and autonomic balance might slow down HRs, which probably contributed to the lengthening of the i-PRi, but had no significant effects on the i-QRSd. Lastly, i-QRSd can change with alteration in the cardiac electrical conduction velocity, which is mediated by the gap junction (GJ), a main channel for intercellular impulse propagation. In HF conditions, down-regulation of the GJ in the ventricular muscle was reportedly associated with the slowing of the myocardial conduction velocity, QRS widening, and contractile dysfunction [21]. Therefore, i-QRSd might be further reduced if the expression or function of the GJ is restored with CRT response. Furthermore, ventricular muscle expresses abundant GJs composed of connexin (Cx) 43, which offers high conductance, whereas the myocytes of the AV conduction system have sparsely dispersed small GJs containing Cx 45, with lower conductance [21]. This differential expression profile of Cx subunits could thus be related to the different electrical remodeling patterns in the i-PRi and i-QRSd following CRT. The recovery kinetics of Cx 43 and Cx 45 might also differ after CRT implantation. Prescription of beta-blockers or amiodarone can also affect the i-QRSd or i-PRi, however, there was no significant change in the proportions of patients who were treated by such medications during the follow-up in our data (Table 4).

### 4.3. Clinical Implications

Prolonged PRi can exert a significant adverse effect on cardiac hemodynamics and long-term clinical outcomes, increasing the risk of AF and all-cause mortality in the general population. In patients with acute and chronic HF, prolonged PRi is associated with a worse prognosis compared with patients with a normal PRi [5,15]. Even, among CRT patients, it was identified as an independent predictor for worse outcomes, and those with prolonged baseline PRi experienced increased all-cause mortality during follow-up.

Initially, we anticipated that i-PRi would decrease with CRT response, at least in the super-responder group, just as a marked narrowing of i-QRSd was found in CRT responders. However, contrary to our expectation, the i-PRi was not shortened even in the CRT super-responders, but rather increased in all groups and increased significantly in CRT non-responders (Figure 2). Therefore, lengthening of the i-PRi along with widening of the i-QRSd might deserve to be investigated or taken as a useful surrogate marker for poor CRT response or the worsening of HF after CRT implantation. Measuring the i-PRi or i-QRSd can be a cumbersome process; however, many cardiac implantable electronic devices are already equipped with algorithms for calculating automatically those intrinsic values [10,12].

On the other hand, the present study suggested that the i-PRi and the i-QRSd might change continuously over time following CRT (Figure 2 and Supplementary Materials Figure S1). Therefore, the optimal AV and VV delays might also change constantly, implying that repetitive re-optimization might be needed to increase the CRT response, especially in non-responders. Indeed, several studies have shown that the optimal AV and VV delays for CRT patients changed significantly during follow-up [8]. In a study of 40 CRT recipients, the optimal AV/VV delays remained unchanged during the follow-up period in only three patients. The mean value of optimal AV delay was extended from 115 ms at baseline to 137 ms at 9 months. Therefore, those authors recommended that the optimal AV/VV delays be regularly reevaluated and reprogrammed [9]. Additionally, recent trials found better clinical outcomes in CRT patients who received continuous automatic re-optimization than in those optimized conventionally with a fixed AV/VV setting in terms of QRS narrowing, CRT response rate, and the risks of death, HF-hospitalization, or AF incidence [10,22,23].

We initially hypothesized that the shortening of the i-PRi with electrical reverse remodeling could allow the new algorithms to be applied to more CRT responders. However, contrary to our hypothesis, none of our CRT patients showed a significant decrease in i-PRi during follow-up. Therefore, the automated AV optimization algorithms might be applicable mainly to patients without markedly prolonged PRi at the baseline. In patients with markedly prolonged baseline i-PRi, atrial lead may need to be implanted interatrial septum, which may be technically challenging, to obtain a relatively short AV delay to get more benefits from automated optimization algorithms of the latest CRT devices.

### 4.4. Limitations

Several limitations of our study need to be acknowledged. First, this is a single-center study with a small number of patients, though our patient group is the largest cohort in whom changes in native conduction properties were investigated with CRT pacing intentionally withdrawn, and we had the longest follow-up duration (33.4 ± 17.9 months) of any relevant study in the literature. Second, differences or changes in the expression patterns of Cx subunits between the AV conduction system and the ventricular muscle were not investigated over the follow-up period or according to the degree of CRT response. Therefore, animal experiments need be performed to verify the mechanisms we proposed to explain our results. Lastly, detailed information on changes in the prescribed drug doses and their effects on impulse propagation velocity were not fully taken into account over the entire follow-up period. However, we evaluated medications at baseline and the final follow up, and additional analyses were performed between subgroups treated with and without beta-blockers and amiodarone.

## 5. Conclusions

LV volume and i-QRSd decreased markedly with CRT response. However, the i-PRi was not shortened even in the CRT super-responders but increased regardless of CRT responsiveness. CRT-induced electrical reverse remodeling might occur preferentially in the intraventricular conduction system but not in the AV conduction system. Repeated optimization of the AV or VV intervals might be needed to maximize CRT response due to ongoing changes in the AV and VV conduction properties after CRT implantation.

## Figures and Tables

**Table 1:** Baseline characteristics.

	Total (*n* = 100)	Non-Responders (*n* = 29)	Responders (*n* = 71)	*p*-Value
**Clinical characteristics**				
Age (year)	65.7 ± 11.5	67.1 ± 11.1	65.0 ± 11.7	0.442
Female gender	40	9 (31%)	31 (44%)	0.242
Body mass index (kg/m^2^)	23.8 ± 3.7	24.3 ± 3.8	23.6 ± 3.6	0.387
Hypertension	62	23 (79%)	39 (55%)	0.023
Diabetes	39	12 (41%)	27 (38%)	0.755
Chronic kidney disease	11	3 (10%)	8 (11%)	0.894
Valvular heart disease	49	17 (59%)	32 (45%)	0.219
Ischemic cardiomyopathy	30	13 (45%)	17 (24%)	0.039
**Echocardiographic parameters**				
LVEF (%)	24 ± 6	23 ± 6	24 ± 5	0.222
LVEDV (mL)	251 ± 66	273 ± 66	242 ± 65	0.029
LVESV (mL)	192 ± 58	212 ± 57	184 ± 57	0.028
**Electrocardiographic parameters**				
Heart rate (b.p.m.)	73 ± 13	72 ± 13	74 ± 13	0.554
PR interval (ms)	199 ± 34	205 ± 35	197 ± 34	0.290
QRS duration (ms)	166 ± 36	157 ± 19	170 ± 41	0.115
QTc interval (ms)	509 ± 43	503 ± 43	512 ± 43	0.336
Left bundle branch block	80	18 (62%)	62 (87%)	0.004
**Medication at discharge**				
Beta blocker	83	21 (72%)	62 (87%)	0.072
ACE inhibitor or ARB	92	25 (86%)	67 (94%)	0.172
Aldosterone antagonist	76	20 (69%)	56 (79%)	0.292
Diuretics	98	28 (97%)	70 (99%)	0.509
Amiodarone	18	9 (31%)	9 (13%)	0.030

Values are presented as mean with standard deviation or number (%).

**Table 2:** Changes in echocardiographic and electrocardiographic parameters.

	Pre-CRT (Baseline)	Post-CRT * (Last Follow-Up)	*p*-Value
**Echocardiographic parameters**			
LVEF (%)	24 ± 6	40 ± 15	<0.001
LVEF in responders	24 ± 5	45 ± 9	<0.001
LVEF in non-responders	23 ± 6	24 ± 5	0.477
LVEDV (mL)	251 ± 66	183 ± 89	<0.001
LVEDV in responders	242 ± 65	140 ± 49	<0.001
LVEDV in non-responders	273 ± 66	288 ± 76	0.040
LVESV (mL)	192 ± 58	122 ± 82	<0.001
LVESV in responders	184 ± 57	81 ± 45	<0.001
*LVESV in non-responders*	*212 ± 57*	*222 ± 65*	*0.084*
**Electrocardiographic parameters (intrinsic values measured during CRT-off mode)**			
QRS duration in all patients (ms)	166 ± 36	160 ± 23	0.125
QRS duration in responders	170 ± 39	159 ± 24	0.012
QRS duration in non-responders	157 ± 19	165 ± 20	0.001
PR interval in all patients (ms)	199 ± 34	205 ± 37	0.035
PR interval in responders	197 ± 34	200 ± 35	0.142
PR interval in non-responders	205 ± 35	219 ± 40	0.005
Heart rate (bpm)	73 ± 13	66 ± 10	<0.001
Heart rate in responders	73 ± 12	67 ± 10	<0.001
Heart rate in non-responders	72 ± 13	64 ± 10	0.005

Values are presented as mean with standard deviation or number (%). * The last echocardiograms and the electrocardiograms recorded closest in time to the last echocardiographic examination were used for the post-CRT values. LVEF*,* left ventricular ejection fraction; LVEDV*,* left ventricular end-diastolic volume; LVESV*,* left ventricular end-systolic volume; QTc interval*,* corrected QT interval.

**Table 3:** Absolute changes in echocardiographic and electrocardiographic parameters according to response status to CRT.

	Non-Responders (*n* = 29)	Responders (*n* = 71)	*p*-Value
Follow-up duration (month)	32.9 ± 19.0	33.6 ± 17.5	0.857
LVEF (%)	1 ± 6	22 ± 13	<0.001
LVEDV (mL)	15 ± 38	−101 ± 58	<0.001
LVESV (mL)	10 ± 30	−103 ± 42	<0.001
Intrinsic heart rate (bpm)	−8 ± 14	−7 ± 14	0.846
Intrinsic PR interval (ms)	14 ± 25	2 ± 24	0.023
Intrinsic QRS duration (ms)	8 ± 13	−11 ± 42	0.014
Paced HR (b.p.m.)	−4 ± 14	−6 ± 16	0.519
Paced PR interval (ms)	−41 ± 39	−33 ± 39	0.355
Paced QRS duration (ms)	−12 ± 27	−40 ± 42	0.002

Values are presented as mean with standard deviation or number (%). Positive values indicate increase whereas negative values decrease in the specific variables. LVEF, left ventricular ejection fraction; LVEDV, left ventricular end-diastolic volume; LVESV, left ventricular end-systolic volume; QTc interval, corrected QT interval.

**Table 4:** Changes in the intrinsic PR interval and medications.

	Pre-CRT (Baseline)	Post-CRT (Last Follow-Up)	*p*-Value *
**Changes in the intrinsic PR interval**			
** Intrinsic PR interval (ms) according to beta-blocker use and CRT responsiveness**			
** **Responder with beta-blocker (*n* = 60)	197 ± 35	200 ± 36	0.153
** **Responder without beta-blocker (*n* = 11)	196 ± 28	200 ± 28	0.413
** **Non-responder with beta-blocker (*n* = 27)	206 ± 36	219 ± 42	0.053
** **Non-responder without beta-blocker (*n* = 2)	190 ± 3	215 ± 7	0.180
** Intrinsic PR interval (ms) according to amiodarone use and CRT responsiveness**			
** **Responder with amiodarone (*n* = 7)	236 ± 57	243 ± 66	0.596
** **Responder without amiodarone (*n* = 64)	192 ± 28	195 ± 26	0.096
** **Non-responder with amiodarone (*n* = 10)	217 ± 33	240 ± 42	0.059
** **Non-responder without amiodarone (*n* = 19)	198 ± 35	208 ± 33	0.190
**Changes in medications**			
** Beta-blocker use**	83	87	0.553
** **Beta-blocker in responder, *n*	62	61	1.000
** **Beta-blocker in non-responder, *n*	21	26	0.505
** Amiodarone use**	18	17	1.000
** **Amiodarone in responder, *n*	9	7	0.795
** **Amiodarone in non-responder, *n*	9	10	1.000

Values are presented as mean with standard deviation or number (%). * Wilcoxon singed rank test *p*-value.

## Supplementary Materials

The following are available online at https://www.mdpi.com/2077-0383/9/7/2152/s1, Figure S1: Serial changes in the intrinsic PR interval and QRS duration during the follow-up period.
