# Supplementary figures and images for: Electrical Reverse Remodeling of the Native Cardiac Conduction System after Cardiac Resynchronization Therapy
Source: J Clin Med. 2020 Jul 8;9(7):2152. doi: 10.3390/jcm9072152 (PMC7408635; doi:10.3390/jcm9072152)

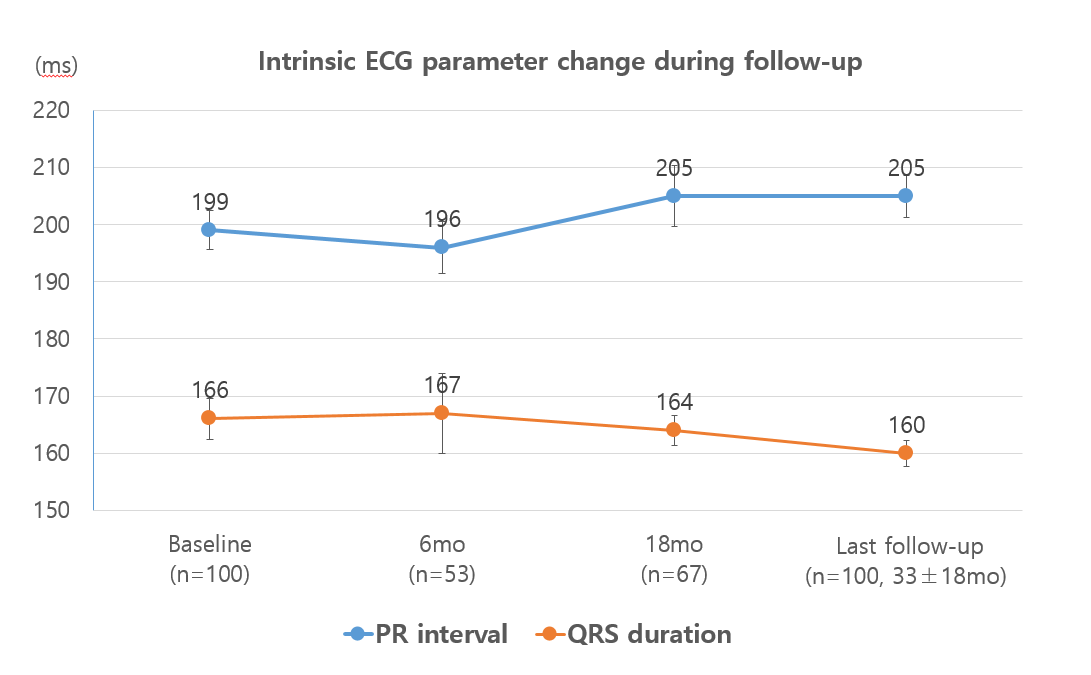

Supplement: Supplementary file 1 [file jcm-09-02152-s001.zip › jcm-835797-SI.tif]
